# Efficacy and safety of endovascular brachytherapy combined with transarterial chemoembolization for the treatment of hepatocellular carcinoma patients with type III or IV portal vein tumor thrombosis

**DOI:** 10.1186/s12957-022-02495-4

**Published:** 2022-02-02

**Authors:** Ling Li, Niangmei Cheng, Xinhui Huang, Xiadi Weng, Yubin Jiao, Jingfeng Liu, Wuhua Guo

**Affiliations:** 1 Mengchao Hepatobiliary Hospital of Fujian Medical University, Fuzhou, 350025 China; 2 The First affiliated hospital of Fujian Medical University, Fuzhou, 350025 China; 3 The First Clinical Medical College of Fujian Medical University, Fuzhou, 350025 China

**Keywords:** Endovascular brachytherapy, Transarterial chemoembolization, Hepatocellular carcinoma, Portal vein tumor thrombosis

## Abstract

**Background:**

The purpose of this study was to evaluate the efficacy and safety of endovascular brachytherapy (EVBT) combined with transarterial chemoembolization (TACE) for the treatment of hepatocellular carcinoma (HCC) complicated with type III OR IV portal vein tumor thrombosis (PVTT) and to further analyze the prognostic predictors for the patients with HCC and PVTT.

**Methods:**

We retrospectively analyzed the medical records of 54 patients who were diagnosed with HCC complicated with type III or IV PVTT and received EVBT combined with modified TACE treatment from January 2017 to June 2019. Adverse events, treatment response, overall survival (OS), progression-free survival (PFS), and stent patency were analysed to evaluate the efficacy and safety of this treatment. The independent prognostic predictors of OS were also statistically analyzed by the cox regression model.

**Results:**

No adverse events occurred in the enrolled patients receiving EVBT combined with TACE treatment. The objective response and disease control rates were 42.6% and 96.3% respectively within 4 weeks after the treatment. The median OS and PFS were 209 days and 138 days, respectively. Cumulative stent patency rate was 70.4% at the last follow-up. AFP ≥ 400 ng/ml, ECOG PS > 1, Child Pugh grade B, and non-hemihepatic HCC were independent risk predictors to evaluate the OS of HCC patient with type III or IV PVTT.

**Conclusions:**

EVBT combined with TACE was a relatively effective and safe strategy to treat HCC patients with type III or IV PVTT.

## Background

Liver cancer is the fourth leading cause of cancer-related mortality globally [1], and ranks second among cancer-related deaths in China [2]. Hepatocellular carcinoma (HCC) is the major histological subtype of liver cancer (accounting for 70–90%) [3]. Portal vein tumor thrombosis (PVTT) is a common complication of HCC, reported to accompany 44–62.2% of HCC [4]. PVTT can lead to portal hypertension and tumor metastasis [5], so as to the prognosis of HCC patients with PVTT is still very poor (overall survival time less than 4 months) [6, 7]. According to clinical practice, Cheng’s classification of PVTT has been proven to be a significant and useful classification system for disease evaluation, treatment selection, and prognostic prediction of HCC with PVTT. According to Cheng’s classification, types III and IV PVTT refer to tumor thrombi involving the main portal vein trunk and superior mesenteric vein, respectively [8, 9], indicating that this is difficult-to-treat advanced stage disease with poor prognosis. Recently, transarterial chemoembolization (TACE), targeted drugs, or yttrium-90 resin microspheres for correctional selective internal radiation therapy were applied to treat the patients who cannot receive surgical resection. Among these therapeutic approaches, TACE exerts its anti-tumor effects by injecting chemotherapeutic agents into the hepatic artery, and thus causes the selective obstruction of the tumor-feeding arteries. Target drugs (such as sorafenib or Lenvatinib), on the other hand, suppress HCC growth, metastasis, and angiogenesis by inhibiting the activity of tyrosine kinase. However, all these approaches only showed limited clinical efficacy in the treatment of HCC complicated with type III PVTT [10–15].

Iodine 125 (^125^I) seeds can continuously release low-energy γ-rays, which can destroy the tumor cells and cause the most destructive damage without endangering normal tissues, thus achieving the purpose of tumor control. Therefore, ^125^I seed implantation for endovascular brachytherapy (EVBT) has been proven to be a local adjuvant radiotherapy treatment with safe and reliable features and can be used for the treatments of multiple cancer types including HCC, pancreatic cancer, non-small-cell lung cancer, and cervical cancer [16–18]. Some evidence on treatment of HCC with type III PVTT indicated that EVBT and TACE combined treatment is an optimized therapeutic strategy that is superior to the single application of TACE or TACE combined with portal vein stent implantation (or external radiotherapy) [19–22]. However, to our knowledge, evaluations of the efficacy and safety of HCC patients with type IV PVTT receiving EVBT and TACE combined treatment have not been performed. Even patients with type III PVTT with invasion of the second intrahepatic portal vein branches were always excluded by previous studies. Therefore, the aim of this study was to evaluate the efficacy and safety of EVBT and TACE combined therapy for the treatment of HCC patients with type III or IV PVTT in Fujian area of China.. Meanwhile, statistical analysis was also performed to screen the independent risk factors for evaluating the prognosis of HCC patients after they received EVBT and TACE combined therapy. Notably, 7 HCC patients (13.0%) with invasion of the second intrahepatic portal vein branches, and 10 HCC patients (18.5%) with type IV PVTT were enrolled in this study, whose prognosis was evaluated for the first time.

## Methods

### Patients

This study was approved by the ethics committee of our institute. Before undergoing treatment, patients and their relatives were informed the benefits of the treatment and possible adverse events were explained to the patients and their relatives in detail, and all patients provided written informed consent to the received treatment.

**Fig. 1:**
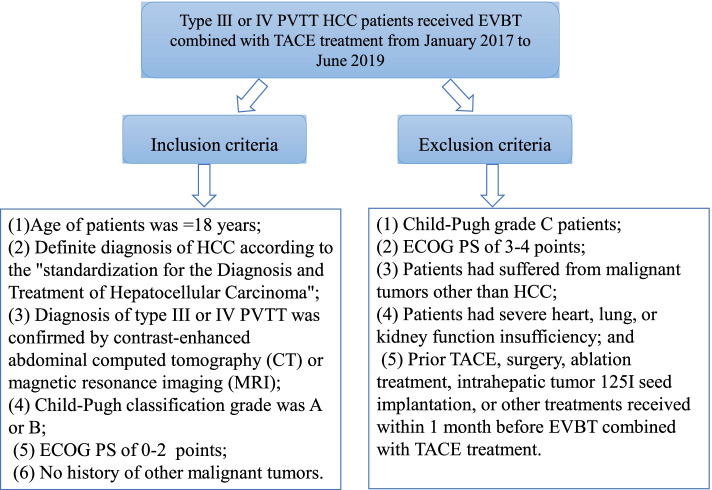
Inclusion and exclusion criteria of HCC patients

The medical records of patients who were diagnosed with HCC complicated with type III or IV PVTT and who received EVBT combined with TACE treatment from January 2017 to June 2019 were retrospectively analyzed. The inclusion criteria for this study were as follows: (1) age of patients was ≥ 18 years; (2) definite diagnosis of HCC according to the “standardization for the Diagnosis and Treatment of Hepatocellular Carcinoma”; (3) diagnosis of type III or IV PVTT was confirmed by contrast-enhanced abdominal computed tomography (CT) or magnetic resonance imaging (MRI); (4) Child-Pugh classification grade was A or B; (5) Eastern Cooperative Oncology Group performance status (ECOG PS) of 0–2; and (6) no history of other malignant tumors. The exclusion criteria were as follows: (1) Child-Pugh grade C patients; (2) ECOG PS of 3-4 points; (3) patients had suffered from other malignant tumors; (4) patients had severe heart, lung, or kidney function insufficiency; and (5) prior to TACE, surgery, ablation treatment, intrahepatic tumor ^125^I seed implantation, or other treatments received within 1 month before EVBT combined with TACE treatment (Fig. 1).

### EVBT treatment

#### Stent and 125I seeds

The nitinol self-expanding stent (Luminxx III, Bard, USA) used in this study was 10–12 mm in diameter and 60–120 mm in length. ^125^I seeds used in this study were a brachytherapy source (HTA, China) with a silver wire in the core, containing ^125^I seeds 3.00 mm in length. The seeds were encapsulated by high-purity titanium 0.8 mm in diameter and 4.5 mm in length. The radioactivity of each ^125^I seed was 0.6–0.7 mCi, with a half-life of 59.43 days, and the principal photon emissions were 31.4 keV X-rays and 35.5 keV γ-rays. Meanwhile, activated silver wire could also emit fluorescent X-rays at 22.1–22.5 keV. The half-value thickness of tissue for ^125^I seeds was 17 mm, and the initial dose rate was 0.0013 Gy/min. These seeds were arranged linearly and sealed into a 4F catheter continuously to construct a ^125^I seed strand. Studies have shown that continuous linearly arranged ^125^I seed strand radiation distribution isometric curve was a cylinder, which was suitable for intracavitary treatment [23–25].

#### Implantation of the portal vein stent and ^125^I seeds

**Fig. 2:**
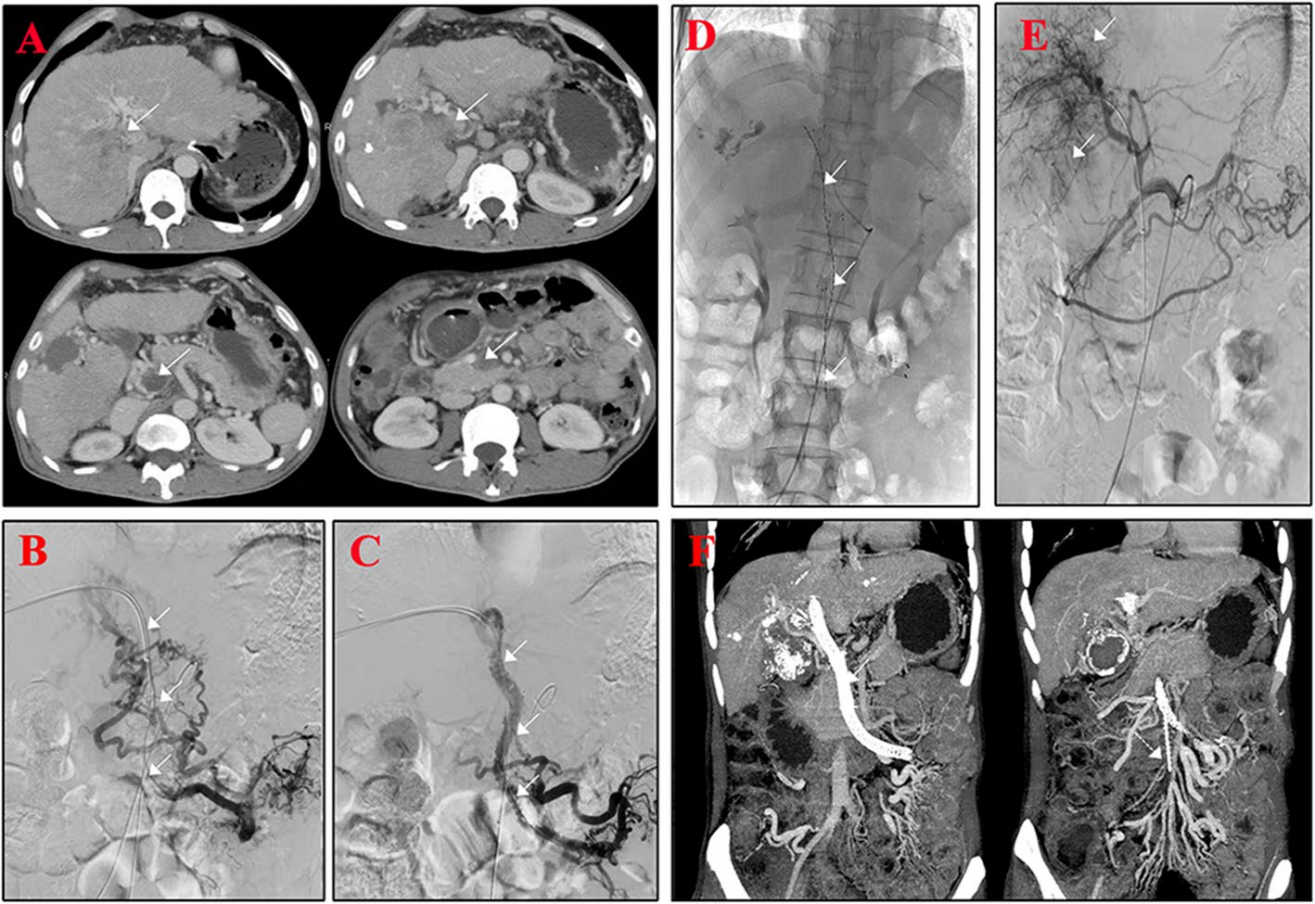
EVBT combined with TACE in patients with HCC with-type IV PVTT. The PVTT involved the superior mesenteric vein in preoperative enhanced CT, as shown by the arrows (**A**). The main portal vein and superior mesenteric vein were filled with tumor thrombus in preoperative superior mesenteric venography (**B**). The superior mesenteric vein and main portal vein could be effectively displayed in postoperative superior mesenteric venography(**C**). The ^125^I seeds were shown in the fluoroscopy after EVBT (**D**). The arrows point to the stained tumor blood vessels in intraoperative angiography of the celiac trunk (**E**). The stent was well placed with good patency (**F**)

Implantation of the portal vein stent and ^125^I seeds were performed before TACE. The first or second branch of the intrahepatic portal vein was punctured with a 22-G Chiba needle (COOK, Bloomington, USA) under ultrasound guidance, and then a 0.018-in wire (COOK) was inserted into the portal vein after removal of the puncture needle core. A 6F NEFF set (COOK) was introduced into the portal vein, and a wire which was 0.035 in in diameter and 150 cm in length was manipulated across the obstructed portal vein into the superior mesenteric vein or splenic vein. The outer cannula of the NEFF set was replaced by a 6F, 55-cm-long sheath (COOK) via the wire. Venography of the splenic vein and superior mesenteric vein was conducted by a gold-labeled catheter (COOK) to determine whether there were significant esophageal gastric varices and to measure the diameter and length of the PVTT. The vein that supplies the esophageal and gastric varices was embolized with a coil (COOK) and/or GLUNRAN 2 (GEM, Viareggio, Italy). If the PVTT was still present at the puncture point of the portal vein, the measured length was the distance from the puncture point to the distal end of the PVTT. The number of ^125^I seeds to be implanted was determined (*N* = length (mm)/4.5 + 4) [19]. Two 0.035-in, 260-cm-long stiff wires (Terumo, Tokyo, Japan) were inserted into the splenic vein or superior mesenteric vein through the 6F vascular sheath. After the sheath was removed, a self-expandable stent of appropriate size and a 6F vascular sheath (55 cm) were introduced into the appropriate location via stiff wires. The ^125^I seed strand was delivered through the 6F 55 cm vascular sheath, the stent was deployed, and the ^125^I seeds were released at the target position. Finally, the intrahepatic portal vein puncture route was blocked with GLUBRAN 2 (GEM, Viareggio, Italy). The process of implantation was shown in Fig. 2.

### TACE treatment

To determine all the feeding arteries of the tumor, angiography of the abdominal trunk and superior mesenteric artery is performed with a 5F RH catheter (Terumo, Tokyo, Japan). Angiography of the left gastric artery, bilateral phrenic artery, right renal artery, and bilateral internal thoracic artery were also performed if necessary. Then, 5-8 mL of lipiodol, 1 mL of polyvinyl alcohol (PVA) foam embolization particles (COOK), 30 mg of epirubicin (Pfizer, New York, USA), and 22–25 mL of ioversol were mixed and emulsified. The target artery was catheterized with a 2.7F microcatheter (Terumo). Under the guidance of digital subtraction angiography (DSA), the target artery was embolized by the mixture. This process is shown in Fig. 2.

### Postoperative treatment and follow-up

All patients were followed up at 4- to 6-week intervals after the combined therapy until death or their last follow-up (before June 30, 2020). Tumor progression and patency of the portal vein stent were evaluated by abdominal CT or MR enhancement, and laboratory tests were performed to evaluate liver and renal function, blood cell count, and coagulation parameters of the patients. After the operation, some patients also received further treatments according to their disease conditions, such as TACE, tumor targeted ablation therapy, ^125^I seed implantation, or surgical resection.

### Evaluation of efficacy and safety

The efficacy of EVBT and TACE combined therapy in the treatment of HCC complicated with type III or IV PVTT was evaluated by overall survival (OS), progression-free survival (PFS), and the modified response evaluation criteria in solid tumors (mRECIST) in this study [26]. OS was defined as the period from patients receiving EVBT combined with TACE treatment to the date of death or the last follow-up. In this study, PFS was defined as the length of time ranging from patient receiving the combined treatment of EVBT and TACE to the progression of HCC including intrahepatic HCC spread, variceal bleeding, or liver function decompensation. According to mRECIST, the treatment efficacy was classified as complete response (CR), partial response (PR), stable disease (SD), and progressive disease (PD). The standard for reporting adverse events induced by EVBT and TACE combined treatment was the Common Terminology Criteria for Adverse Events (Version 5.0), which was a widely accepted and applied in cancer clinical trials [27–29].

### Statistical analysis

Data were analyzed with SPSS version 20.0 (SPSS, Chicago, Illinois). Continuous variables are presented as the mean value ± standard deviation. OS and PFS were analyzed with Kaplan–Meier curves and log-rank tests. *p* value < 0.05 was regarded as significant. The OS and PFS were shown with median data. The cox regression analysis was performed to determine independent risk factors for overall survival. The variables with *p* < 0.05 in the univariate analysis were selected for multivariate analysis.

## Results

### Patient data

A total of 54 patients were enrolled in this study. Male patients accounted for the majority (*n* = 52, 96.3%), with a mean age of 51 ± 8.2 years (27–68 years). There were 52 patients (96.3%) with cirrhosis caused by hepatitis B, 42 patients (77.8%) with Child-Pugh grade A, and 12 patients (22.2%) with Child-Pugh grade B. Only 5 (9.3%) of these patients had a single tumor, and the largest tumor diameter was > 10 cm in 50% of patients. The tumors of 30 patients (55.6%) were confined to the hemihepatic region. There were 44 patients (81.5%) with type III PVTT and 10 patients (18.5%) with type IV PVTT, and the mean diameter and length of the PVTT were 1.9 ± 0.6 cm and 3.8 ± 2.5 cm, respectively. Fourteen (25.9%) patients had distant metastasis of HCC, and 14 patients (25.9%) had received previous therapies including surgery, TACE, ablation, or radiotherapy more than 1 month earlier before enrollment. Postoperative local therapy for HCC was performed in 36 patients (66.7%), among whom 32 patients received TACE, 14 patients received ^125^I seed implantation, 4 patients received intrahepatic tumor ablation, 2 patients received radiotherapy, and 2 patients (3.7%) underwent tumor resection. The baseline characteristics of these 54 patients are summarized in Table 1 and Table 2.

**Table 1:** Baseline characteristics of the patients

Characteristics	Number (proportion,%)	Characteristics	Number (proportion,%)
Sex		Hemihepatic HCC^c^	
Male	52 (96.3)	Yes	30 (55.6)
Female	2 (3.7)	No	24 (44.4)
Age (years)		Distant metastasis	
≥ 55	18 (33.3)	Yes	14 (25.9)
< 55	36 (66.7)	No	40 (74.1)
Hepatitis B		PVTT^d^	
Yes	52 (96.3)	Type III	44 (81.5)
No	2 (3.7)	Type IV	10 (18.5)
AFP^a^ ( ng/ml)		Tumor diameter	
≥400	29 (53.7)	> 10 cm	27 (50.0)
<400	25 (46.3)	≤ 10 cm	27 (50.0)
Child-Pugh grade		Number of tumors	
A	42 (77.8)	Single	5 (9.3)
B	12 (22.2)	Multiple	49 (90.7)
ECOG PS^b^		Previous treatment	
0/1	42 (77.8)	Yes	14 (25.9)
2	12 (22.2)	No	40 (70.5)
Hepatic arterioportal fistulas		Postoperative treatment	
Yes	34 (73.0)	Yes	36 (66.7)
No	20 (37.0)	No	18 (33.3)

^a^*AFP* alpha fetoprotein

^b^*ECOG PS* Cooperative Oncology Group performance status

^c^*HCC* hepatocellular carcinoma

^d^*PVTT* portal vein tumor thrombosis

**Table 2:** Characteristics of PVTT

Characteristic	Number (Proportion%)
Unilateral first branch invasion	20 (37.0)
Left portal vein invasion	6 (11.1)
Right portal vein invasion	14 (25.9)
Unilateral secondary branches invasion	20 (37.0)
All unilateral secondary branches invasion	16 (29.6)
Bilateral first branch invasion	34 (63.0)
Unilateral secondary branches invasion	11 (20.4)
Bilateral secondary branches invasion	23 (42.6)
All bilateral secondary branches invasion	7 (13.0)

### Safety analysis

No complications related to the implantation of EVBT with ^125^I seeds, such as abdominal bleeding, stent displacement, white blood cell decline caused by ^125^I seeds, or radiation damage to surrounding organs, were observed during treatment. The most common adverse events were post-chemoembolization syndrome, including fever, vomiting, liver pain, liver function damage, pleural effusion, and primary peritonitis. Grade 3 adverse events, such as primary peritonitis (3.7%) and postoperative increases in TBIL (29.6%) or ALT (9.3%), were observed, as presented in Table 3. The TBIL and ALT almost returned to the preoperative level one month after the treatment. There were no grade 4 adverse events in the treatment.

**Table 3:** Adverse treatments events

Adverse events	Number (proportion%)	Grade 1–2, *n* (proportion%)	Grade 3, *n* (proportion%)	Grade 4, *n* (proportion%)
Abdominal pain	26 (48.1)	26 (48.1)	0 (0)	0 (0)
Fever	23 (42.6)	23 (42.6)	0 (0)	0 (0)
Vomiting	7 (13.0)	7 (13.0)	0 (0)	0 (0)
Ascites	6 (11.1)	6 (11.1)	0 (0)	0 (0)
Pleural effusion	5 (9.3)	5 (9.3)	0 (0)	0 (0)
TBIL^a^ increase	28 (81.5)	28 (51.9)	16 (29.6)	0 (0)
ALT^b^ increase	15 (27.8)	10 (18.5)	5 (9.3)	0 (0)
Hypoalbuminemia	43 (79.6)	43 (79.6)	0 (0)	0 (0)
Inguinal hematoma	0 (0)	0 (0)	0 (0)	0 (0)
Gastrointestinal bleeding	0 (0)	0 (0)	0 (0)	0 (0)
Pulmonary embolism	0 (0)	0 (0)	0 (0)	0 (0)
Primary peritonitis	2 (3.7)	0 (0)	2 (3.7)	0 (0)

^a^*TBIL* total bilirubin

^b^*ALT* alanine aminotransferase

### Efficacy analysis

#### Responses of HCC

The responses of HCC were evaluated according to mRECIST 4 weeks after the treatment. During the 4-week treatment, no cases with CR or PVTT progression were observed within the coverage of ^125^I seeds. The PR rate and SD rate of the enrolled patients were 42.6% and 53.7% respectively.

#### Overall survival

Forty-four of 54 enrolled patients (81.5%) eventually died in this study. Among the dead patients, 42 participants died of liver failure (95.5%). One patient died of lung infection and one patient died of esophageal and gastric variceal bleeding. The median survival time was 209 days. The cumulative survival rates of all enrolled patients at 180, 360, 720, and 1080 days were 56%, 28%, 16%, and 12%, respectively. The cumulative survival rates of patients with type IV PVTT at 180, 360, 720, and 1080 days were 70%, 30%, 20%, and 20%, respectively.

Next, cox regression model was conducted to analyze the important prognostic factors of overall survival OS. The results showed that AFP (*p* = 0.045), ECOG PS (*p* = 0.008), Child-Pugh grade (*p* = 0.033), ascites (*p* = 0.014), and hemihepatic HCC (*p* = 0.011) were significantly associated with the OS of patients in the univariate analysis, which was selected for multivariate analysis. Subsequently, the multivariate Cox regression analysis indicated that AFP ≥ 400 ng/ml, ECOG PS > 1, Child-Pugh grade B, and non-hemihepatic HCC (HCC located in both the left and right hemiliver) were independent risk predictors of OS for the treatment of EVBT combined with TACE in our study (Table 4). Additionally, in the multivariate analysis, there is no HR interval values for ascites due to no significance of it. Unexpectedly, PVTT classification was not an independent predictor of OS. Two reasons may explain this result. Firstly, the liver function of patients with type IV PVTT was kept at normal level before enrollment. Furthermore, we found that these patients had formed the hepatic portal vein collateral circulation before they received combined treatment, which maintained blood supply instead of the portal vein. Therefore, enrolled patients with type IV PVTT have a better prognosis, indicating that PVTT is not an independent predictor of OS. It also indicated that EVBT and TACE combined treatment might be beneficial for patients with type IV PVTT but having normal liver function and formation of hepatic portal vein collateral circulation.

**Table 4:** Risk predictors of OS in the univariate and multivariate analyses

Variable	Overall survival			
	Univariate analysis		Multivariate analysis	
	HR (95% CI)	*P* value	HR (95% CI)	*P* value
Age (≥55 years old vs < 55 years old)	1.247 (0.673–2.310)	0.483		
AFP^a^ (≥400ng/ml vs < 400 ng/ml)	1.858 (1.014–3.404)	0.045^*^	1.940 (1.008–3.735)	0.047^*^
Ascites (Have vs None)	2.15 (1.171–3.948)	0.014^*^		0.067
ECOG PS^b^ (0–1 vs. 2)	2.414 (1.230–4.740)	0.008^*^	2.574 (1.278–5.185)	0.008^*^
Child-Pugh grade (A vs B)	2.114 (1.061–4.214)	0.033^*^	2.913 (1.385–6.123)	0.005^*^
Arterioportal fistula (Have vs None)	1.036 (0.555–1.931)	0.912		
Hemihepatic HCC^c^ (Yes vs. No)	0.454 (0.247–0.835)	0.011^*^	0.517 (0.269–0.992)	0.047^*^
HCC^c^ diameter (>10cm vs. ≤ 10 cm)	1.454 (0.802–2.636)	0.218		
Number of tumors (Single vs. Multiple)	0.327 (0.100–1.260)	0.065		
Distant metastasis (Yes vs. No)	1.829 (0.955–3.502)	0.069		
Hepatic vein tumor thrombosis (Yes vs. No)	1.844 (0.850–4.003)	0.122		
Cavernous transformation collateral (Yes vs. No)	0.703 (0.387–1.275)	0.246		
PVTT^d^ type (III vs. IV)	0.801 (0.371–1.731)	0.573		

^*^P < 0.05

^a^*AFP* alpha fetoprotein

^b^*ECOG PS* Cooperative Oncology Group performance status

^c^*HCC* hepatocellular carcinoma

^d^*PVTT* portal vein tumor thrombosis

**Fig. 3:**
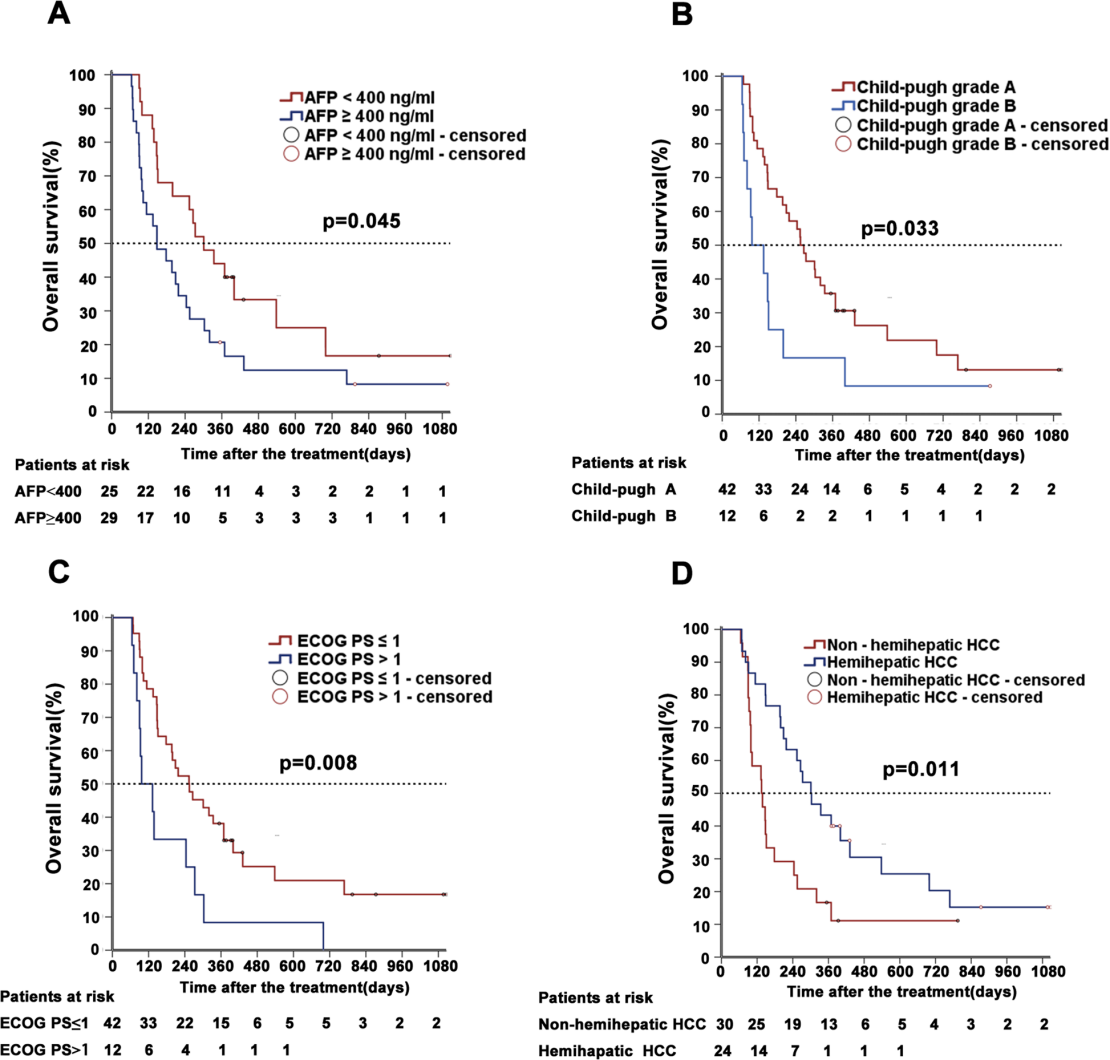
Kaplan–Meier analysis for overall survival. Kaplan-Meier survival curves of OS between AFP ≥ 400 ng/ml and AFP < 400 ng/ml (**A**). Child Pugh grade A and Child Pugh grade B (**B**); ECOG PS>1 and ECOG PS≤1 (**C**); non-hemihepatic HCC and hemihepatic HCC (D)

In addition, Kaplan-Meier analysis was applied to evaluate the survival of enrolled patients. All patients (*n* = 54) were further divided into two subgroups depending on different prognostic indicators, namely, AFP (≥ 400 ng/ml vs. < 400 ng/ml), ECOG PS (> 1 vs. ≤ 1), Child Pugh grade (grade A vs. grade B), or non-hemihepatic HCC (Yes vs. No). The results showed that patients with high serum AFP (≥ 400 ng/ml, *n* = 29) usually have shorter survival time compared to those with low serum AFP (*p* = 0.045, Fig. 3A). The median OS time of the patients with high and low serum AFP was 148 and 301 days, respectively. Similarly, Child Pugh grade B (*p* = 0.033), ECOP PS > 1 (*p* = 0.008), and non-hemihepatic HCC (*p* = 0.011) were also significantly associated with the poor prognosis (Fig. 3B, C, D).

#### Progression-free survival

In our results, the median progression-free survival (PFS) time of all enrolled patients was 138 days. The cumulative PFS ratio was 56%, 35%, 15%, and 2% at 90, 180, 360, and 720 days, respectively, after treatment.

#### Stent patency

DSA data showed the stent patency in 40 patients (74.0%) just immediately after ^125^I and stent implantation, and 42 patients (77.7%) had stent patency in the follow-up 1 month after the treatment. The cumulative stent patency rate was 70.4% (*N* = 38).

## Discussion

Although the OS of HCC patients has been improved due to the implementation of a variety of effective therapies in recent decades, the prognosis of HCC patients with PVTT, especially with type III or IV PVTT, is still very poor [30], which has become a hot topic in clinical research to further improve the therapeutic efficacy of HCC patients.

According to Chinese guideline, TACE, systemic treatment (such as sorafenib or lenvatinib), and surgical resection could be selected to treat HCC patients with PVTT. They have been proved to be effective for treating these patients [31–33]. However, based on clinical experiences, two or more treatment approaches are always simultaneously applied to treat HCC patients with PVTT, and harvest a better prognosis than single use of any one approach. For examples, patients with advanced HCC received the combination treatment of TACE and sorafenib usually harvested better prognosis than those only treated with either approach alone [34]. Sorafenib as an adjuvant treatment of HCC patients who underwent resection was helpful to further prolong OS and RFS [35]. However, any one of these combinations can be used, the MSTs (median survival time) of HCC patients with type III PVTT remained less than 7 months and median RFS after surgery in HCC patients with combined type III PVTT was only 1.61 months [4, 12, 15, 36–38]. This suggests that there is still treatment improvement and optimization of the existing combination therapy approach. A recent study showed that the efficacy of radiotherapy combined with TACE was better than that of TACE or TACE combined with sorafenib in treating HCC patients with type III PVTT [39]. However, it was reported that the MST of the patients receiving EVBT and TACE combined treatment was approximately 11.7 months, which was longer than that of radiotherapy combined with TACE (9.5 months) when treating HCC patients with type III PVTT [22]. To our knowledge, the reasons why EVBT and TACE combined treatment exhibited better efficacy could be attributed to the following points: (1) compared with other treatments, EVBT combined with TACE could restore portal vein perfusion of the liver more quickly and improve the liver function of patients; (2) continuous radiotherapy with ^125^I seeds in EVBT elicits an anti-intimal hyperplasia effect [40] and can improve stent patency [41, 42]; (3) EVBT can effectively inhibit the progression of PVTT, reduce the intrahepatic spread of HCC caused by PVTT, and maximumly retain the healthy liver [21]; and (4) TACE treatment can effectively restrain the tumor growth [43].

This study showed that the MST of HCC patients with type III OR IV PVTT treated with EVBT and TACE combined therapy was 7.0 months, which was longer than the MST of patients treated with TACE, sorafenib, or TACE and sorafenib combined therapy. However, the OS of enrolled patients in this study was shorter than the patients enrolled in other studies focusing on EVBT combined with TACE for the treatment of HCC patients with PVTT (7.0 months vs. 9.3–11.7 months) [21, 22]. This was mainly because the enrolled patients in our study suffered from more advanced disease than the patients enrolled in previously published reports. For example, there were 10 patients (18.5%) with HCC complicated with type IV PVTT and 34 patients (63%) with invaded main portal veins and bilateral primary branches in this study. In addition, the tumors of 27 patients (50%) were > 10 cm, with only 5 patients (13%) having tumors < 5 cm. Whereas in previous studies, patients with HCC complicated with type IV PVTT were excluded, and the proportion of patients (32% or 36% vs. 13%) who had tumors < 5 cm was higher in that in our study [21, 22]. Therefore, it was reasonable that the MST of our enrolled patients was shorter than the previously reported MST. There was no significant difference in OS between patients with type III and type IV PVTT in our study. Among the 10 patients with type IV PVTT, there were 7 patients with Child-Pugh grade A and 3 patients with Child-Pugh grade B. Only one patient had HCC with diameter larger than 10 cm, 9 patients had hemihepatic HCC, and 10 patients had good portal collateral vessels supplying the liver. Exhibiting lower tumor burden and hemihepatic HCC in the patients with type IV PVTT may be the reasons why there was a lack of a significant difference in OS between patients with type III and type IV PVTT.

According to our results, EVBT combined with TACE is an exciting and promising therapeutic strategy for the treatment of HCC patients with type III or IV PVTT. This study was a single-center retrospective study, and the enrolled patients received different postoperative treatments which limited us to harvest more valuable results. Therefore, to further confirm these results, a multi-center clinical research with larger cohorts should be performed in the future.

The embolization method of TACE was also modified in our study to further improve its efficacy. Conventional TACE (c-TACE) formulation of ethiodized oil (such as Lipiodol®) and gelatin sponge (such as Gelfoam®) had drawbacks on patient tolerance and resulted in undesired systemic toxicity [44]. Furthermore, more than half of the enrolled patients (*n* = 34, 63.0%) had arterioportal fistulas, which could further increase false embolism. In this study, ethiodized oil mixed with PVA particles (with the diameter of 500 to 710 μm) was used for embolization to ensure efficacy of TACE and prevent lipiodol from entering the portal vein system of healthy liver tissues through the arterioportal fistula. The results showed that our modified approach dramatically improved the disease control rate to 96.3% within 1 month after TACE treatment. Therefore, we believed that this approach can be valuable for EVBT combined with TACE therapy, and further work is required to confirm these findings.

In this study, the length of ^125^I seed chain was confirmed depending on the distance from the puncture point on the healthy side (or the relatively healthy side) to the distal end of the PVTT. An ^125^I seed chain was placed to cover the whole distance to suppress PVTT-mediated HCC progression and reduce the risk of liver function getting worse. In addition, most of the ^125^I seeds were released on one side of the vessel. Our results indicated that no progression of PVTT was observed in all enrolled patients treated with ^125^I seeds. Furthermore, no adverse events were observed during ^125^I seed treatments, indicating that eccentric ^125^I seeds inoculation was an effective approach to suppress the development of PVTT.

## Conclusions

In the present study, a combined therapeutic strategy with EVBT and TACE has been developed and applied in clinical practice. This combination therapy might be an effective and safe strategy to improve the prognosis of HCC patients with type III or IV PVTT according to our results. Additionally, AFP ≥ 400 ng/ml, ECOG PS > 1, Child-Pugh grade B, and non-hemihepatic HCC were proved to be independent risk factors to predict the OS of HCC patients with type III or IV PVTT.

## Data Availability

The datasets generated during and/or analyzed during the current study are available from the corresponding author on reasonable request.

## Abbreviations

CT: Contrast-enhanced abdominal computed tomography
CR: Complete response
DSA: Digital subtraction angiography
EVBT: Endovascular brachytherapy
ECOG PS: Eastern Cooperative Oncology Group performance status
HCC: Hepatocellular carcinoma
^125^I: Iodine 125
MST: Median survival time
MRI: Magnetic resonance imaging
mRECIST: Modified response evaluation criteria in solid tumors
OS: Overall survival
PVTT: Portal vein tumor thrombosis
PFS: Progression-free survival
PR: Partial response
PD: Progressive disease
PVA: Polyvinyl alcohol
SD: Stable disease
TACE: Transarterial chemoembolization
